# The Relationship Between Anxiety and Suicidal Ideation in Patients With Bipolar Disorder: Chain Mediation Effects of Social Support and Self-Esteem

**DOI:** 10.62641/aep.v53i6.2035

**Published:** 2025-12-17

**Authors:** Yan Lu, Chenxia Song, Juan Chen, Long Wang, Yang Du, Xinqiong Zhang

**Affiliations:** ^1^College of Nursing, Anhui Medical University, 230032 Hefei, Anhui, China; ^2^Department of Emotional Disorders, The Fourth People’s Hospital of Hefei, 230022 Hefei, Anhui, China

**Keywords:** bipolar disorder, suicide, suicidal ideation, social support, self concept, mediation analysis

## Abstract

**Background:**

Bipolar disorder is associated with a high prevalence of suicidal ideation, and comorbid anxiety may further increase suicide risk. The psychological mechanisms linking anxiety to suicidal ideation, especially the roles of social support and self-esteem, remain unclear. This study constructed a chain mediation model to examine how anxiety, social support and self-esteem relate to suicidal ideation in patients with bipolar disorder.

**Methods:**

From March 2022 to May 2023, 450 inpatients with bipolar disorder were recruited from six hospitals in Anhui Province. Standardised scales assessed anxiety, social support, self-esteem and suicidal ideation. Pearson correlation examined associations between variables, binary logistic regression identified factors associated with suicidal ideation, and Hayes’ PROCESS macro tested the chain mediation model.

**Results:**

Logistic regression showed that higher anxiety increased the odds of suicidal ideation, whereas higher social support and self-esteem reduced the odds. Suicidal ideation was positively correlated with anxiety and negatively correlated with social support and self-esteem, and mediation analysis demonstrated a significant direct effect of anxiety and significant independent and chain mediating effects of social support and self-esteem (all *p* < 0.001).

**Conclusions:**

Anxiety is related to suicidal ideation in patients with bipolar disorder both directly and indirectly through reduced social support and self-esteem. The indirect pathway via self-esteem showed the largest effect, underscoring the need to enhance social support and self-esteem in suicide prevention for this population.

## Introduction

Bipolar disorder (BD) is a chronic mental illness characterised by alternating or mixed episodes of mania or hypomania and depression [1]. About 30% of people with BD attempt suicide [2]. Suicide usually occurs in three stages: ideation, attempt and action [3]. Suicidal ideation is a precursor to suicidal behaviour and the first step of such behaviour [4]. It is also the top five most effective predictors of future suicide deaths [5] and plays a predictive role in suicide attempts.

Anxiety symptoms are highly prevalent in BD (lifetime prevalence ~42.7%) [6]. Given that anxiety often co-exists with BD and is clinically linked to increased distress, we examined whether this co-occurrence relates to elevated suicidal ideation and attempts in BD compared with non-BD populations. On this basis, we hypothesise that anxiety positively predicts suicidal ideation in hospitalised patients with BD.

Suicidal ideation in patients with BD results from the interaction of complex physiological and socio-psychological factors. In patients with depression, heightened anxiety symptoms correlate with a deteriorated suicide experience. One study demonstrated that depression and anxiety are significantly and positively related to the incidence of non-suicidal self-injury [7]. A survey investigating mental health trends among Norwegian students from 2010 to 2023 revealed a significant increase in psychological distress and suicidal ideation [8], which may be due to the fact that students face stressors such as academic pressure, financial insecurity and social disconnection [9]. Patients with BD commonly have comorbid anxiety symptoms, and the occurrence of non-suicidal self-injury is closely related to anxiety symptoms [10]. Therefore, this study proposes Hypothesis 1: Anxiety positively predicts suicidal ideation in hospitalised patients with BD.

Social support refers to the perceived or tangible assistance provided by an individual’s social network, which includes emotional and informational support [11]. It plays an important role in reducing suicidal ideation in people with BD. Studies have shown that high social support corresponds to a reduced likelihood of suicidal ideation in women [12], and perceived social support serves as a crucial mediator between expressive suppression and suicidal ideation [13]. Therefore, this study proposes Hypothesis 2: The degree of social support plays a mediating role between anxiety symptoms and suicidal ideation.

Self-esteem is an individual’s overall positive evaluation of the self [14] and is a protective factor for mental health. An international study showed that low self-esteem is directly related to depression and suicidal ideation [15]. The stigmatisation of patients with BD leads to low self-esteem [16]. This diminished self-esteem may be associated with loss of confidence in life, poor adaptability to the environment and suicidal ideation. Thus, this study proposes Hypothesis 3: Self-esteem plays a mediating role between anxiety and suicidal ideation in hospitalised patients with BD.

Social support and self-esteem influence each other. The higher a person’s perceived social support, the higher their self-esteem [17]. People with high self-esteem are more effective in establishing and maintaining social relationships and may receive more social support than those with low self-esteem [18]. This study proposes Hypothesis 4: Self-esteem and social support play a cascading mediating role between anxiety and suicidal ideation in hospitalised patients with BD. Grounded in connectedness-to-self-worth processes, we specified the a priori chain as follows: ‘anxiety → diminished perceived social support → corresponds to reduced self-esteem → increased suicidal ideation’. In BD, perceived social connectedness is theorised to underpin self-worth, thereby placing social support ahead of self-esteem in the chain. We recognise potential bidirectionality (e.g., low self-esteem may inhibit social behaviour and diminish perceived support); given the cross-sectional design, the chain reflects theory-consistent ordering rather than a causal claim. On the basis of the above background and hypothesis, the early identification of suicidal ideation must be strengthened in patients with BD. Therefore, this study, based on the guidance of the Bipolar Suicide Model (BSM) [19], explores the interplay of anxiety, social support, self-esteem and suicidal ideation in hospitalised patients with BD and reveals its underlying mechanism by developing a chain mediation model. This study is based on the BSM, which defines suicidality as the result of the interaction of threat/entrapment assessments, cognitive vulnerability and ‘rescue’ factors. Within this framework, anxiety heightens danger assessment, self-esteem reflects resilience to appraisal and perceived social support measures connectedness/rescue that mitigates the progression from conceptualisation to action. Accordingly, we hypothesised that anxiety would be positively associated with suicidal ideation, with perceived social support and self-esteem serving as partial, and potentially, serial mediators of this relationship. The prevention of suicide in patients with BD has important practical implications and can provide theoretical support and decision-making guidance for relevant clinical departments.

All variables included in this study are included in the BSM framework, and no additional constructs have been introduced. Within this model, anxiety and suicidal ideation are categorised as emotional components, self-esteem is treated as a coping strategy and social support is considered part of the rescue system.

## Method

### Study Samples

This is a cross-sectional study. From March 2022 to May 2023, patients with BD hospitalised in Hefei Fourth People’s Hospital, Lu’an Second People’s Hospital, Anqing Sixth People’s Hospital, Huangshan Second People’s Hospital, Xuancheng Fourth People’s Hospital and Feidong County Third People’s Hospital were selected as research subjects by convenient sampling. The inclusion criteria were as follows: (1) fulfilment of the diagnostic criteria for BD according to the International Classification of Diseases and Related Health Problems, Tenth Edition (ICD-10); (2) adherence to a stable regimen of antipsychotics and/or mood stabilisers for ≥4 weeks without dose change >25%, and clinically stable/remitted as defined by a Clinical Global Impression–Severity (CGI-S) ≤3 at enrolment, maintained for the preceding 4 weeks, with no ICD-10 current manic/hypomanic/depressive episode recorded [20] (Note: HAMA was collected as a study variable and not used to define stability.); (3) age between 18 and 65 years, regardless of gender; (4) patients with at least a primary school education and with sufficient reading and comprehension skills to complete the scale assessment required for the study; and (5) informed consent to participate in the study and to accept the relevant assessments, requiring the signature of the patient or legal guardian. The exclusion criteria were as follows: (1) patients with neurological diseases, other mental illnesses or substance dependence; and (2) patients unable to complete the measurement of various scales for other reasons. We restricted inclusion to medicated patients in stable or remission phases (≥4 weeks) to ensure reliable self-reporting across multiple scales and minimise the confounding effects of acute mood symptoms or medication initiation. Acute-phase or drug-naïve patients were excluded because their unstable clinical status and high symptom burden would compromise assessment validity and ethical feasibility in this cross-sectional survey.

This multi-centre study was conducted in accordance with the Declaration of Helsinki and received ethical approval from the Institutional Review Board of Anhui Mental Health Center (Ref: HFSY-IRB-PJ-LY [2021003]), which served as the central ethics committee overseeing all participating institutions. Additionally, each collaborating hospital’s ethics committee provided local approval **Supplementary Material** (**Supplementary Table 1**). All participants or their legal guardians provided written informed consent.

### Sample Size Calculation 

Our primary analysis was a serial mediation specified as an observed-variable path model equivalent to PROCESS Model 6, implemented using Hayes’ PROCESS macro (Model 6; www.processmacro.org) in IBM SPSS Statistics (version 26.0, IBM Corp., Armonk, NY, USA).We conducted an a priori Monte Carlo simulation (Mplus v8.0; 20,000 replications) using small-to-moderate path coefficients (a/b paths ≈ 0.20–0.25), yielding an expected total indirect effect ≈ 0.10. At Cronbach’s α = 0.05, the minimum sample size to achieve ≥80% power was N = 280. Allowing ~7% attrition, the target N = 300; we ultimately enrolled N = 450, exceeding the target. As a conservative cross-check, a simple ‘≥20 observations per free path coefficient’ heuristic yields ≈240, which was also surpassed by our achieved N.

### Data Collection

General Questionnaire: The team designed the questionnaire based on the literature review, including gender, age, religious belief, marital status, education, family income, place of residence, course of illness, family history and presence of suicidal factors. The following standardised instruments were used:

(1) Suicidal Ideation Self-Rating Scale (SIOSS). Developed by Xia and Wang [21], the SIOSS comprises 26 items across four dimensions: hopelessness, optimism, sleep disturbance and concealment. The Chinese version demonstrated good internal consistency (Cronbach’s α = 0.79) in clinical samples. The SIOSS had a Cronbach’s α of 0.80, which was consistent with reliability indices reported in similar Chinese clinical samples. Items were scored 0/1, with higher totals indicating greater suicidal ideation. For binary analyses, presence of suicidal ideation was defined as SIOSS total ≥12 and concealment subscale <4; records with concealment ≥4 were considered invalid according to the scale manual and excluded from the binary outcome. The continuous SIOSS total was used in correlation and mediation analyses.

(2) Hamilton Anxiety Rating Scale (HAMA). In this study, we employed the Chinese version of the HAMA translated and culturally adapted by Wang *et al*. [22] (1993). This 14-item scale assesses somatic and psychic anxiety. Validation in Chinese psychiatric inpatients yielded a Cronbach’s α of 0.91. In the present sample, Cronbach’s α was found to be approximately 0.86, consistent with previous findings in Chinese psychiatric populations, indicating good internal consistency.

(3) Rosenberg Self-Esteem Scale (SES) [23]. We used the Chinese adaptation of the Rosenberg SES by Ji and Yu (2005), consisting of 10 items rated on a 4-point Likert scale (with six reverse-scored items). The Chinese version has shown acceptable reliability (Cronbach’s α = 0.81) in adolescent populations. The internal consistency of the SES in this sample was acceptable, with Cronbach’s α was determined to be 0.90, comparable to other Chinese psychiatric cohorts.

(4) Social Support Rating Scale (SSRS) [24]. Developed by Xiao [24], the SSRS contains 10 items covering three dimensions: subjective support, objective support and support utilisation. It has demonstrated satisfactory internal consistency (Cronbach’s α = 0.82) in Chinese community samples. Cronbach’s α in this study was found to be 0.83, in line with prior research on Chinese psychiatric outpatients, demonstrating satisfactory reliability.

### Survey Methods and Quality Control

This study used face-to-face interviews to collect data. All investigators followed a unified SOP and scripted interview manual; they received centralised training and certification (post-test ≥90%, inter-rater ICC ≥0.80). Standardisation included version-controlled materials, randomised scale order and EpiData eCRFs with range/logic checks; a coordinating centre maintained a site query log with a72-hour resolution to minimise collection bias.

All sites followed a unified SOP and rater manual. Investigators underwent centralised training and certification (post-test ≥90% and inter-rater reliability ICC ≥0.80), with quarterly refreshers. A coordinating centre held weekly calls, maintained a site query log with a 72-hour resolution and supervised EpiData eCRFs with range checks, dual entry and audit trails. High-risk findings triggered immediate senior review and adherence to IRB-compliant reporting.

### Statistical Methods

Chain mediation was assessed using an observed-variable path model equivalent to PROCESS Model 6, employing bias-corrected bootstrap (5000 resamples) for the inference of indirect effects. Epidata software (version 3.1; EpiData Association, Odense, Denmark; www.epidata.dk) was used for data entry. IBM SPSS Statistics (version 24.0; IBM Corp., Armonk, NY, USA) was used for the statistical analysis of the subjects’ general and clinical data. Variables adhering to a normal distribution were presented as mean ± SD and compared by independent-samples *t*-test or one-way ANOVA, as appropriate. Variables with non-normal distribution were reported as median (interquartile range) and compared between two groups using the Mann–Whitney U test or among multiple groups using the Kruskal–Wallis H test. Count data were expressed as frequencies and percentages, and the chi-squared test was used for comparison between groups. Binary logistic regression was used to analyse the factors influencing suicidal ideation in patients with BD. All variables with significant differences in univariate analysis were incorporated into the regression model. In addition, variables considered clinically relevant or theoretically important were retained in the model to control for potential confounders, regardless of their statistical significance. Pearson correlation analysis was performed to analyse the relationship between the scores of each scale. The PROCESS programme developed by Hayes was used to test for chain mediation effects. The treatment variables were initially standardised, and Model 6 in PROCESS 3.4 was used to test the value of the chain mediation effect of social support and self-esteem between anxiety symptoms and suicidal ideation. The bias-corrected percentile bootstrap method (5000 repeated samples) was used to test the above pathways. *p* < 0.05 was considered statistically significant.

In multivariable logistic regression, we included variables associated with suicidal ideation at *p* < 0.10 in univariate analyses, along with predetermined clinical/psychological covariates (anxiety, perceived social support and self-esteem). Employment/education status was dummy coded with “employed/enrolled” as the reference group and “leave-of-absence/break” as the exposure category. The binary outcome (suicidal ideation: yes/no) for logistic regression was constructed using the aforementioned SIOSS criteria (total ≥12 with concealment <4; concealment ≥4 treated as invalid and excluded). To mitigate common method bias (CMB), we implemented procedural controls (anonymity, standardised instructions and randomised scale order) and conducted Harman’s single-factor test; the first unrotated factor accounted for <40% of the total variance, indicating no serious CMB.

## Results

### Basic Information of the Survey Subjects

Among the 450 patients, 155 (34.44%) exhibited suicidal ideation, including 275 males and 175 females. The age range was from 18 years old to 65 years old, with an average age of 35.2 ± 12.0. The mean HAMA score was 18.54 ± 3.02, the mean SSRS score was 33.43 ± 8.93, the mean SES score was 29.57 ± 5.57 and the mean SIOSS score was 13.06 ± 3.49.

### Univariate Analysis of Suicidal Ideation

Significant differences were observed between the groups in terms of gender, age, employment/education status, history of suicide or self-harm, presence of suicidal triggers, HAMA score, SSRS score and SES score (*p* < 0.05). Further details are presented in Table 1.

**Table 1.** **Single-factor analysis of suicidal ideation**.

Content of the survey		Suicidal ideation		χ^2^/t value	*p* value
		No (n = 295)	Yes (n = 155)		
Sex				60.092	<0.001
	Male	219 (79.6)	56 (20.4)		
	Female	76 (43.4)	99 (56.6)		
Age (years)		37.12 ± 12.05	31.47 ± 11.35	4.821	<0.001
Religion				0.362	0.547
	Yes	84 (67.7)	40 (32.3)		
	No	211 (64.7)	115 (35.3)		
Marital status				3.832	0.429
	Unmarried	155 (68.6)	71 (31.4)		
	Married without children	22 (59.5)	15 (40.5)		
	Married with children	93 (64.6)	51 (35.4)		
	Divorced	21 (55.3)	17 (44.7)		
	Widowed	4 (80.0)	1 (20.0)		
Educational attainment				0.830	0.842
	Primary school	29 (64.4)	16 (35.6)		
	Junior high school	74 (63.8)	42 (36.2)		
	High school or junior college	94 (68.6)	43 (31.4)		
	College or above	98 (64.5)	54 (35.5)		
Household Income				3.509	0.320
	<30,000	74 (59.7)	50 (40.3)		
	30–50 thousand	89 (67.9)	42 (32.1)		
	50,000–100,000	70 (64.8)	38 (35.2)		
	More than 100,000	62 (71.3)	25 (28.7)		
Employment/Education				7.670	0.006
	Yes	168 (71.5)	67 (28.5)		
	No	127 (59.1)	88 (40.9)		
Place of residence				0.7911	0.374
	Urban	98 (62.8)	58 (37.2)		
	Rural	197 (67.0)	97 (33.0)		
Family history				0.000	0.995
	None	257 (65.6)	135 (34.4)		
	Yes	38 (65.5)	20 (34.5)		
History of suicide and self-injury				11.781	0.001
	None	268 (68.5)	123 (31.5)		
	Yes	27 (45.8)	32 (54.2)		
Suicide triggers				5.573	0.018
	None	279 (67.1)	137 (32.9)		
	Yes	16 (47.1)	18 (52.9)		
HAMA total score		13.66 ± 3.40	27.83 ± 2.12	47.269	<0.001
SSRS total score		35.52 ± 8.79	29.45 ± 7.79	7.228	<0.001
SES total score		30.82 ± 5.08	27.18 ± 5.72	6.894	<0.001
SIOSS total score		9.65 ± 3.30	19.55 ± 3.82	28.615	<0.001

**Note: **Employment/education status coded dichotomously: ‘Yes’ = employed or actively enrolled; ‘No’ (reference) = unemployed, discontinued schooling, or on leave/school break (inactive). Suicide triggers: ‘Yes’ = major life-change events within 6 months; ‘No’ (reference) = no such events. Suicidal ideation (binary) defined by SIOSS total ≥12 with concealment <4; concealment ≥4 deemed invalid. **Abbreviations: **HAMA, Hamilton Anxiety Rating Scale; SSRS, Social Support Rating Scale; SES, Self-Esteem Scale; SIOSS, Suicidal Ideation Self-Rating Scale.

### Pearson Correlation Analysis

The results of correlation analysis indicated a significant positive correlation between the SIOSS score and the HAMA score, as well as a significant negative correlation between the SIOSS score and the SSRS and SES scores. Further details are presented in Table 2.

**Table 2.** **Correlation analysis results of HAMA, SSRS, SES and SIOSS in patients with bipolar disorder (r value)**.

Variables	HAMA	SSRS	SES	SIOSS
HAMA	1.000	–	–	–
SSRS	–0.202^a^	1.000	–	–
SES	–0.378^a^	0.387^a^	1.000	–
SIOSS	0.329^a^	–0.323^a^	–0.310^a^	1.000

**Note: **Pearson r (two-tailed). ^a^*p* < 0.05. **Abbreviations: **HAMA, Hamilton Anxiety Rating Scale; SSRS, Social Support Rating Scale; SES, Self-Esteem Scale; SIOSS, Suicidal Ideation Self-Rating Scale.

### Regression Analysis of Factors Influencing Suicidal Ideation

Binary logistic regression analysis was conducted with the presence or absence of suicidal ideation (coded as 1 for suicidal ideation and 0 for no suicidal ideation) as the dependent variable and the variables exhibiting differences in general data and scale scores as the independent variables. Employment/education status was coded as a dichotomous variable: participants were classified as ‘Yes’ if they were employed or enrolled in school immediately prior to hospital admission, and ‘No’ (reference category) if they were unemployed at home or had discontinued schooling. Suicide triggers were similarly coded dichotomously: ‘Yes’ indicates presence of major life-change events within the six months before admission, and ‘No’ (reference category) indicates absence of such events. This coding mirrors approaches used in prior suicide risk research on socioeconomic factors. Suicide precipitants were defined based on the SRRS, with major life-changing events occurring within the six months before admission serving as reference for precipitating stressors. The results demonstrated that gender, age, disease course, anxiety, social support and self-esteem were significant predictors of suicidal ideation in patients with BD (*p* < 0.05), as illustrated in Table 3. Although employment/education status, history of suicide and self-injury and suicide triggers did not reach statistical significance in the regression model (*p* > 0.05), they were retained because of theoretical assumptions and clinical considerations. This approach was adopted to adequately control for potential confounding factors and enhance the robustness and generalisability of the findings. Although employment/education status showed a significant crude association with suicidal ideation in univariate analysis (Table 1), this effect diminished and was no longer significant after adjusting for anxiety, perceived social support, self-esteem and other covariates (Table 3; *p* = 0.560). This pattern suggested that the bivariate association was largely accounted for by the proximal psychological factors included in the multivariable model.

**Table 3.** **Regression analysis of factors influencing suicidal ideation in patients with bipolar disorder (Reference categories indicated in parentheses)**.

Independent variable	SE	B	Exp(B) (95% CI)	*p*-value
Gender	0.269	1.831	6.238 (3.684, 10.565)	<0.001
Age	0.013	–0.059	0.943 (0.919, 0.967)	<0.001
Employment/education	0.256	0.149	1.161 (0.703, 1.917)	0.560
Disease duration	0.016	0.047	1.048 (1.015, 1.082)	0.004
History of suicide self-injury	0.421	0.616	1.851 (0.811, 4.223)	0.144
Suicide triggers	0.510	–0.444	0.641 (0.236, 1.743)	0.384
HAMA total score	0.016	0.058	1.060 (1.026, 1.060)	<0.001
SSRS total score	0.016	–0.075	0.928 (0.899, 0.957)	<0.001
SES total score	0.028	–0.105	0.901 (0.852, 0.952)	<0.001

**Note: **Employment/education status: reference group is ‘No’ (unemployed at home or discontinued schooling). Suicide triggers: reference group is ‘No’ (no major life-change events within 6 months prior to admission). Suicidal ideation (binary) defined by SIOSS total ≥12 with concealment <4; concealment ≥4 deemed invalid. **Abbreviations: **HAMA, Hamilton Anxiety Rating Scale; SSRS, Social Support Rating Scale; SES, Self-Esteem Scale; SE, Standard Error; CI, Confidence Interval.

### Chain Mediation Test of Social Support and Self-esteem Between Anxiety Symptoms and Suicidal Ideation in Patients With Bipolar Disorder

**Fig. 1.**
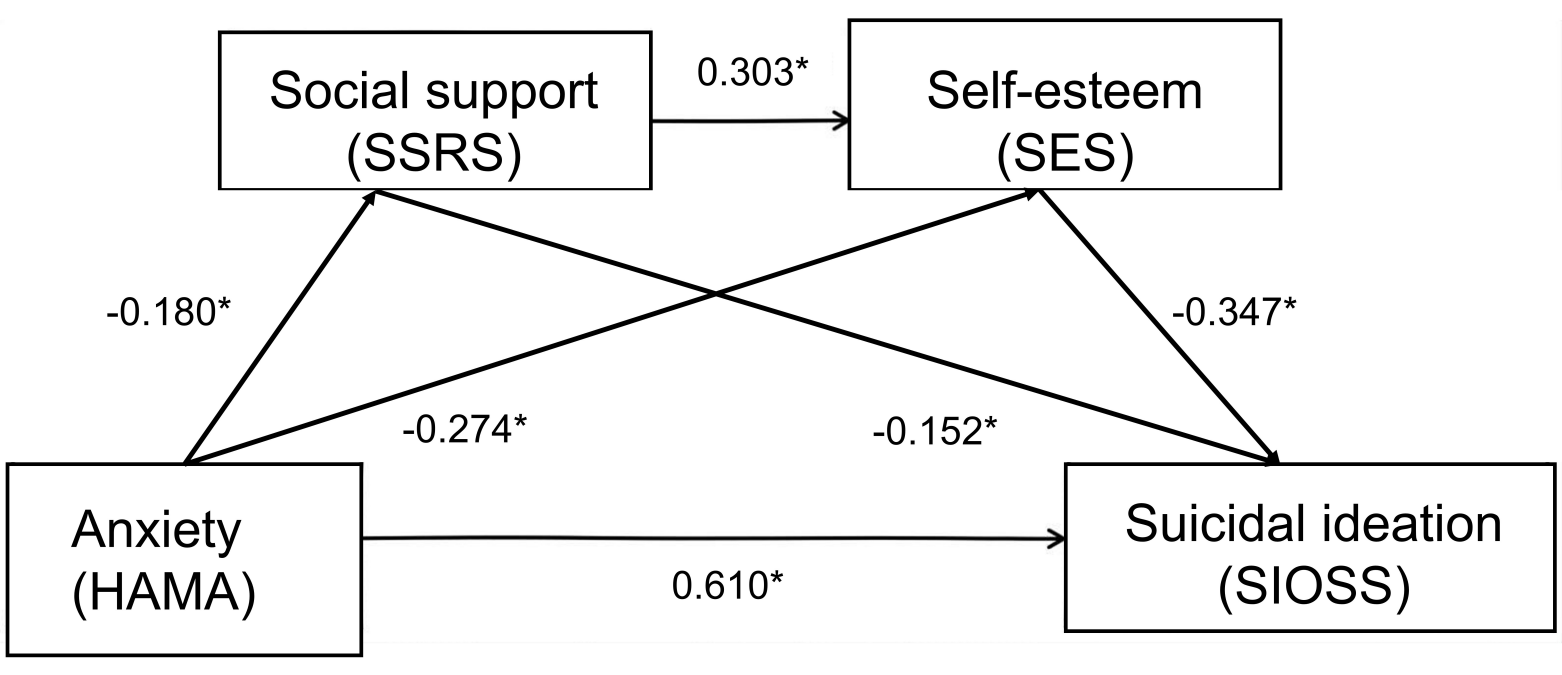
**Chain mediation model of social support and self-esteem between anxiety and suicidal ideation in patients with bipolar disorder**. *p < 0.05. SSRS, Social Support Rating Scale; SES, Self-Esteem Scale; SIOSS, Suicidal Ideation Self-Rating Scale; HAMA, Hamilton Anxiety Rating Scale.

To further explore the relationship among anxiety, social support, self-esteem and suicidal ideation, the present study employed chain mediation modelling analysis (Table 4). The schematic of the mediating effect model is shown in Fig. 1. After correcting for potential confounders such as age, gender, employment/education status, history of suicidal self-injury and suicidal triggers, the results showed that anxiety had a direct positive effect on suicidal ideation (β = 0.601, t = 15.736, *p* < 0.01), whereas anxiety was indirectly associated with suicidal ideation through social support and self-esteem.

**Table 4.** **Chain mediation model analysis**.

Regression equation*		Overall fit index			Significance			Model	
Outcome variable	Predictive variable	R^2^	Adjusted R^2^	F value	β value	t value	*p* value	SE	Coefficients
Social support	Anxiety	0.055	0.042	4.302	−0.180	−3.727	<0.01	0.051	−0.191
Self-esteem	Anxiety	0.309	0.298	28.211	−0.274	−6.528	<0.01	0.028	−0.181
	Social support				0.303	7.455	<0.01	0.025	0.189
Suicidal ideation	Anxiety	0.411	0.403	51.533	0.610	15.736	<0.01	0.023	0.359
Suicidal ideation	Anxiety	0.557	0.548	69.178	0.459	13.008	<0.01	0.021	0.274
	Social support				−0.152	−4.381	<0.01	0.02	−0.086
	Self-esteem				−0.347	−9.093	<0.01	0.035	−0.314

Corrected for age, gender, employment/education status (ref: No), history of suicide self-injury and suicide triggers (ref: No). *: Regression equations are based on Hayes’ PROCESS macro Model 6 (observed-variable path analysis for chain mediation). All predictor variables were standardized before analysis. **Abbreviations: **SE, Standard Error.

### Mediation Effect Test

Mediated effect analysis showed that the total effect of anxiety on suicidal ideation was 0.359, of which 76.32% was a direct effect (β = 0.274, t = 13.008, *p* < 0.01) and 23.68% was a mediated effect (Table 5). The mediating effect was further decomposed into three paths: path 1 (anxiety → social support → suicidal ideation) accounted for 4.46% (β = 0.016, Boot 95% CI: 0.011–0.047), path 2 (anxiety → self-esteem → suicidal ideation) accounted for 15.88% (β = 0.057, Boot 95% CI: 0.016–0.133) and path 3 (anxiety → social support → self-esteem → suicidal ideation) accounted for 3.34% (β = 0.012, Boot 95% CI: 0.008–0.031). Notably, the chained path (Path 3) accounted for only 3.34% of the total effect (β = 0.012), indicating a statistically detectable yet small magnitude compared with the independent self-esteem path (15.88%). Given the absence of a consensus on a minimal clinically significant proportion for indirect effects and the lack of a pre-specified criterion in our protocol, we interpret the chained path as complementary rather than central to the overall pattern. These results suggested that anxiety not only directly increased suicidal ideation but also indirectly increased the risk of suicidal ideation by decreasing social support and self-esteem.

**Table 5.** **Chain-mediated model paths and effect sizes**.

Effect	Path	Effect value	Boot standard error	Boot 95% CI lower limit	Boot 95% CI upper limit	Proportion of effect
Total effect		0.359	0.023	0.314	0.404	100.00%
Direct effect		0.274	0.021	0.233	0.316	76.32%
Mediating effect	Total mediating effect	0.085				23.68%
	Path 1	0.016	0.009	0.011	0.047	4.46%
	Path 2	0.057	0.019	0.016	0.133	15.88%
	Path 3	0.012	0.006	0.008	0.031	3.34%

Path 1: Anxiety → Social support → Suicidal ideation; Path 2: Anxiety → Self-esteem → Suicidal ideation; Path 3: Anxiety → Social support → Self-esteem → Suicidal ideation. Proportion of effect = indirect (or direct) effect divided by the total effect (0.359). Path-specific proportions indicate statistical magnitude rather than clinical thresholds. Given that Path 3 proportion was 3.34% (β = 0.012), it should be interpreted as small; clinical relevance requires replication. **Abbreviations: **CI, Confidence Interval.

## Discussion

In this study, the detection rate of suicidal ideation among inpatients with BD was 34.44%. This rate was broadly consistent with international reports on BD samples (e.g. prevalence ~36% of youth reported suicidal ideation with BD [25]; pooled prevalence ~44%–48% by sex [26]). This high detection rate not only reflects the serious mental health problems faced by patients with BD but also highlights the burden they face. The study also found that suicidal ideation in people with BD is influenced by many factors, including the patient’s age, gender, level of education, history of suicide and self-harm and suicide triggers. Single-factor analysis of the general information sheet showed that the incidence of suicidal ideation was significantly higher among female patients and those on holiday/study than among male patients and those engaged in work/study. In addition, factors such as patients’ anxiety level, social support and self-esteem were significantly related to suicidal ideation. Further chain mediation analysis showed that anxiety symptoms may be directly associated with patients’ suicidal ideation, as well as with suicidal ideation through social support and self-esteem. Anxiety symptoms were also associated with suicidal ideation through the chain mediation effect of social support and self-esteem, indicating that all three hypotheses in this study are valid. From a clinical standpoint, the indirect association via self-esteem (Path 2: 15.88%) was the most substantial among the indirect paths and therefore more relevant for treatment planning, whereas the chained path (Path 3: 3.34%) should be viewed as having limited significance and primarily hypothesis generation. Accordingly, interventions prioritising self-esteem may yield enhanced clinical value in BD populations. Targeted approaches include the integration of structured self-esteem modules into cognitive–behavioural therapy, strength-based behavioural activation and stigma-reduction psychoeducation, complemented by support-enhancing strategies (e.g. family psychoeducation or peer support) and routine anxiety management. These priorities were aligned with the observed magnitude pattern of indirect paths (Path 2 > Path 1 > Path 3).

In multivariable models, female gender, young age and prolonged disease duration remained independently associated with suicidal ideation. Female predominance may reflect a heightened burden of anxiety and responsiveness to interpersonal stress in BD. Young age likely signifies increased impulsivity/affective instability early in the illness. A long duration may encompass cumulative episodes and lingering symptoms. Education level and prior suicide/self-harm were not independent after adjustment, suggesting that their effects partly functioned through anxiety, self-esteem and social support pathways. However, a history of self-harm remains a clinically significant risk marker that warrants regular screening.

The results of mediation analysis showed that anxiety symptoms can directly predict suicidal ideation. The severity of anxiety symptoms was positively correlated with the intensity of suicidal ideation. Anxiety significantly influences suicidal ideation, which is a serious clinical problem in patients with BD. Comorbid anxiety symptoms are very common in people with BD. Almost all anxiety symptoms increase the risk of suicide [27]. The proportion of comorbid anxiety symptoms is high. Patients experience increased perceived distress and frustration, frequently accompanied with elevated suicidal ideation and attempts [28, 29].

Anxiety influences suicidal ideation by inducing social support. Social support is negatively correlated with anxiety symptoms and suicidal ideation; specifically, an increase in social support corresponds to a reduction in anxiety symptoms [30] and a diminished prevalence of suicidal ideation. This was consistent with prior research demonstrating that social support acts as a protective factor against suicidal ideation in patients with cancer [31]. Our chain mediation analysis showed that anxiety was negatively associated with the level of social support, which partially mediated the relationship between anxiety and suicidal ideation. Therefore, interventions targeting anxiety symptoms may improve perceived social support, which corresponds to a reduced likelihood of suicidal ideation in patients with BD. This study showed no evidence for the reverse pathway (i.e. social support influencing anxiety); thus, our discussion is limited to the model-supported direction.

Self-esteem is another mediating variable in the relationship between anxiety and suicidal ideation in patients with BD. This study found that self-esteem was negatively correlated with suicidal ideation, which was consistent with the findings of a previous study [32]. Low self-esteem has been identified as a risk factor associated with anxiety [33]. As the symptoms of anxiety become pronounced, self-esteem diminishes. People with low self-esteem frequently struggle to acknowledge and define their identities, resulting in a diminished sense of agency and suicidal ideation. The investigation into the mechanisms of anxiety and suicidal ideation revealed a chain mediation effect involving social support and self-esteem, which was consistent with a previous study’s conclusions that social support and self-esteem directly influence suicidal ideation [34]. Terror Management Theory suggests that a deficiency in social approval, alongside diminishing self-esteem, may be correlated with depression, despair and a perception that life lacks value, thereby increasing the risk of suicide [35]. Social support can activate an individual’s positive qualities to adapt to maladaptive environments, improve self-esteem and assist patients in managing social relationships. Positive social ties can improve levels of social support, thereby alleviating anxiety symptoms and reducing suicidal ideation. The degree of external family support and the level of internal high self-esteem interact to diminish the prevalence of suicidal ideation.

Anxiety may reduce perceived social support through behavioural and cognitive pathways: behaviourally, heightened anxiety often leads to social withdrawal and diminished help-seeking, thereby limiting opportunities for emotional and instrumental support; cognitively, people with social anxiety disorder may develop negative evaluation expectations (e.g. ‘overestimating threats in situations’) and struggle to handle ongoing social tasks (such as conversations), leading to missed important social cues [36]. Concurrently, anxiety diminishes self-esteem by amplifying negative self-evaluations; persistent worry and rumination foster feelings of personal inadequacy and reduced self-worth. At the neurobiological level, chronic anxiety is associated with dysregulation of the hypothalamic–pituitary–adrenal (HPA) axis, and its severity is correlated with the hyperactivation of relevant brain regions, including the right inferior parietal lobe, the left ventrolateral prefrontal cortex (during emotion processing tasks) and the left posterior superior temporal sulcus and temporoparietal junction (during emotional cognitive control tasks) [37], which is linked to low self-esteem [38]. Consequently, diminished social support and self-esteem significantly mediate the relationship between anxiety [39] and suicidal ideation [40].

This study demonstrated that social support and self-esteem modulate the relationship between anxiety and suicidal ideation in hospitalised patients with BD. This finding offers a novel perspective for healthcare professionals and highlights the need for increased attention to patients’ emotional changes, especially in monitoring and addressing anxiety, promoting adequate social support from family members and prioritising patient care. The diminished significance for employment/education status after adjustment was consistent with a distal-to-proximal pathway: academic/employment disruption may correspond to reduced perceptions of social connectedness and self-worth while exacerbating anxiety, thereby increasing suicidal ideation. In our BSM-grounded framework, these proximal psychological dimensions account for a significant portion of the variance associated with employment/education status in univariate analyses.

The innovation of this study is its focus on inpatients with BD who are at an elevated risk of suicide. From the unique perspective of the chain-mediating role of social support and self-esteem, we aimed to reveal the relationship between different influencing factors to enhance the specificity of the results and practical significance, providing a novel way of understanding the complexity of suicidal ideation. The limitation of this study is its use of a cross-sectional survey, so it was not able to follow long-term changes in suicidal ideation in patients. To enhance understanding of the factors of suicidal ideation in patients with BD, to develop an accurate suicide risk prediction model and to effectively manage those at risk, future studies should use longitudinal follow-up survey methods. This will improve our understanding of changes in patients’ mental states and provide effective support to prevent and correspond to reduced likelihood of suicidal behaviour. This multi-centre study had a relatively small sample size, which limits the precision of estimates, particularly for subgroup analyses. Confirmation in large cohorts is warranted. Another limitation of this study was the exclusion of pharmacological treatment as a covariate in the model construction process. Among patients with BD, pharmacological treatment (such as mood stabilisers and anti-anxiety drugs) is one of the core methods of clinical intervention. During the follow-up period, this treatment method may potentially compromise the assessment results of suicidal ideation due to changes in the concentration of residual drugs in patients’ bodies. Future longitudinal or cross-lagged studies in BD populations, with pharmacological treatment included as a covariate, are needed to elucidate the reciprocal effects of social support and self-esteem. Additionally, given that most variables were self-reported, residual CMB cannot be completely excluded; accordingly, future studies using multi-source assessments are warranted.

## Conclusions

Social support and self-esteem play cascading mediating roles between anxiety and suicidal ideation in patients with BD. In the future, by establishing a risk prediction model for suicidal ideation in patients with BD, early assessment, early identification and timely treatment of high-risk suicidal individuals can be carried out to prevent and control suicide incidents in a timely manner.

## Availability of Data and Materials

All experimental data included in this study can be obtained by contacting the corresponding author if needed.

## Supplementary Material

Supplementary material associated with this article can be found, in the online version, at https://doi.org/10.62641/aep.v53i6.2035.

## References

[1] Leichsenring F, Fonagy P, Heim N, Kernberg OF, Leweke F, Luyten P (2024). Borderline personality disorder: a comprehensive review of diagnosis and clinical presentation, etiology, treatment, and current controversies. *World Psychiatry: Official Journal of the World Psychiatric Association (WPA)*.

[2] Tondo L, Pompili M, Forte A, Baldessarini RJ (2016). Suicide attempts in bipolar disorders: comprehensive review of 101 reports. *Acta Psychiatrica Scandinavica*.

[3] Klonsky ED, May AM (2015). The three-step theory (3ST): a new theory of suicide rooted in the “ideation-to-action” framework. *International Journal of Cognitive Therapy*.

[4] Zhu X, Tian L, Huebner ES (2019). Trajectories of Suicidal Ideation from Middle Childhood to Early Adolescence: Risk and Protective Factors. *Journal of Youth and Adolescence*.

[5] Franklin JC, Ribeiro JD, Fox KR, Bentley KH, Kleiman EM, Huang X (2017). Risk factors for suicidal thoughts and behaviors: A meta-analysis of 50 years of research. *Psychological Bulletin*.

[6] Nabavi B, Mitchell AJ, Nutt D (2015). A Lifetime Prevalence of Comorbidity Between Bipolar Affective Disorder and Anxiety Disorders: A Meta-analysis of 52 Interview-based Studies of Psychiatric Population. *EBioMedicine*.

[7] Wang TW, Gong J, Wang Y, Liang Z, Pang KL, Wang JS (2023). Differences in Non-suicidal Self-injury Behaviors between Unipolar Depression and Bipolar Depression in Adolescent Outpatients. *Current Medical Science*.

[8] Sivertsen B, Skogen JC, Petrie KJ, O’Connor RC, Knudsen AKS, Kirkøen B (2025). Mental health and suicidal ideation from 2010 to 2023 among university students: national repeated cross-sectional analysis. *The British Journal of Psychiatry: the Journal of Mental Science*.

[9] Pedrelli P, Nyer M, Yeung A, Zulauf C, Wilens T (2015). College Students: Mental Health Problems and Treatment Considerations. *Academic Psychiatry: the Journal of the American Association of Directors of Psychiatric Residency Training and the Association for Academic Psychiatry*.

[10] Chen XC, Xu JJ, Yin XT, Qiu YF, Yang R, Wang ZY (2024). Mediating role of anxiety and impulsivity in the association between child maltreatment and lifetime non-suicidal self-injury with and without suicidal self-injury. *Journal of Affective Disorders*.

[11] Holt-Lunstad J, Smith TB, Layton JB (2010). Social relationships and mortality risk: a meta-analytic review. *PLoS Medicine*.

[12] Manning KJ, Chan G, Steffens DC, Pierce CW, Potter GG (2021). The Interaction of Personality and Social Support on Prospective Suicidal Ideation in Men and Women With Late-Life Depression. *The American Journal of Geriatric Psychiatry: Official Journal of the American Association for Geriatric Psychiatry*.

[13] Gupta S, Fischer J, Roy S, Bhattacharyya A (2024). Emotional regulation and suicidal ideation-Mediating roles of perceived social support and avoidant coping. *Frontiers in Psychology*.

[14] Renger D, Reinken A, Krys S, Gardani M, Martiny SE (2023). Why the belief in one’s equal rights matters: Self-respect, depressive symptoms, and suicidal ideation in Western and non-Western countries. *Health Psychology Open*.

[15] Luciano M, Steardo L, Sampogna G, Caivano V, Ciampi C, Del Vecchio V (2021). Affective Temperaments and Illness Severity in Patients with Bipolar Disorder. *Medicina (Kaunas, Lithuania)*.

[16] Zhu T, Kou R, Hu Y, Yuan M, Yuan C, Luo L (2023). Dissecting clinical and biological heterogeneity in clinical states of bipolar disorder: a 10-year retrospective study from China. *Frontiers in Psychiatry*.

[17] Zhou X, Wu X, Zhen R (2018). Self-esteem and hope mediate the relations between social support and post-traumatic stress disorder and growth in adolescents following the Ya’an earthquake. *Anxiety, Stress, & Coping*.

[18] Zhang R (2015). INTERNET DEPENDENCE IN CHINESE HIGH SCHOOL STUDENTS: RELATIONSHIP WITH SEX, SELF-ESTEEM, AND SOCIAL SUPPORT. *Psychological Reports*.

[19] Malhi GS, Bargh DM, Kuiper S, Coulston CM, Das P (2013). Modeling bipolar disorder suicidality. *Bipolar Disorders*.

[20] Guy W (1976). *ECDEU Assessment Manual for Psychopharmacology, Revised (DHEW Publ No ADM 76-338)*.

[21] Xia CY, Wang DB (2010). Development and validation of the Suicidal Ideation Self-Report Scale (SIOSS). *Chinese Mental Health Journal*.

[22] Wang X, Zhang Y, Liu Z (1993). Reliability and validity of the Chinese version of the Hamilton Anxiety Scale in psychiatric patients. *Chinese Mental Health Journal*.

[23] Ji CP, Yu XX (2005). Psychometric evaluation of the Chinese Rosenberg Self-Esteem Scale in adolescents. *Psychological Development and Education*.

[24] Xiao SY (1994). Theoretical basis and research application of the Social Support Rating Scale. *The Journal of Clinical Psychiatry*.

[25] Sewall CJR, Goldstein TR, Salk RH, Merranko J, Gill MK, Strober M (2020). Interpersonal Relationships and Suicidal Ideation in Youth with Bipolar Disorder. *Archives of Suicide Research: Official Journal of the International Academy for Suicide Research*.

[26] Hu FH, Jia YJ, Zhao DY, Fu XL, Zhang WQ, Tang W (2023). Gender differences in suicide among patients with bipolar disorder: A systematic review and meta-analysis. *Journal of Affective Disorders*.

[27] Teng Z, Zhang Y, Wei Z, Liu M, Tang M, Deng Y (2023). Internet addiction and suicidal behavior among vocational high school students in Hunan Province, China: A moderated mediation model. *Frontiers in Public Health*.

[28] Simon NM, Pollack MH, Ostacher MJ, Zalta AK, Chow CW, Fischmann D (2007). Understanding the link between anxiety symptoms and suicidal ideation and behaviors in outpatients with bipolar disorder. *Journal of Affective Disorders*.

[29] Buckner JD, Lemke AW, Jeffries ER, Shah SM (2017). Social anxiety and suicidal ideation: Test of the utility of the interpersonal-psychological theory of suicide. *Journal of Anxiety Disorders*.

[30] Yue C, Liu C, Wang J, Zhang M, Wu H, Li C (2021). Association between social support and anxiety among pregnant women in the third trimester during the coronavirus disease 2019 (COVID-19) epidemic in Qingdao, China: The mediating effect of risk perception. *The International Journal of Social Psychiatry*.

[31] Du L, Shi HY, Qian Y, Jin XH, Li Y, Yu HR (2021). Association between social support and suicidal ideation in patients with cancer: A systematic review and meta-analysis. *European Journal of Cancer Care*.

[32] Wang X, Qiao Y (2022). Parental Phubbing, Self-Esteem, and Suicidal Ideation among Chinese Adolescents: A Longitudinal Mediational Analysis. *Journal of Youth and Adolescence*.

[33] Guil R, Gómez-Molinero R, Merchan-Clavellino A, Gil-Olarte P, Zayas A (2019). Facing Anxiety, Growing Up. Trait Emotional Intelligence as a Mediator of the Relationship Between Self-Esteem and University Anxiety. *Frontiers in Psychology*.

[34] Ingram M, Thorne E, Letourneau EJ, Nestadt PS (2025). Self-Esteem, Perceived Social Support, and Suicidal Ideation and Behavior Among Adults Attracted to Children. *Omega*.

[35] Lieberman EJ (2004). Terror management theory. *The American Journal of Psychiatry*.

[36] Roth DA, Heimberg RG (2001). Cognitive-behavioral models of social anxiety disorder. *The Psychiatric Clinics of North America*.

[37] Díaz DE, Russman Block SR, Becker HC, Phan KL, Monk CS, Fitzgerald KD (2025). Neural Substrates of Emotion Processing and Cognitive Control Over Emotion in Youth Anxiety: An RDoC-Informed Study Across the Clinical to Nonclinical Continuum of Severity. *Journal of the American Academy of Child and Adolescent Psychiatry*.

[38] Pruessner JC, Lord C, Meaney M, Lupien S (2004). Effects of self-esteem on age-related changes in cognition and the regulation of the hypothalamic-pituitary-adrenal axis. *Annals of the New York Academy of Sciences*.

[39] Bdier D, Mahamid F, Fallon V, Amir M (2023). Posttraumatic stress symptoms and postpartum anxiety among palestinian women: the mediating roles of self-esteem and social support. *BMC Women’s Health*.

[40] Seo EH, Yang HJ, Kim SG, Yoon HJ (2022). Ego-resiliency moderates the risk of depression and social anxiety symptoms on suicidal ideation in medical students. *Annals of General Psychiatry*.

