# Supplementary material for: The Relationship Between Anxiety and Suicidal Ideation in Patients With Bipolar Disorder: Chain Mediation Effects of Social Support and Self-Esteem
Source: Actas Esp Psiquiatr. 2025 Dec 17;53(6):1354–65. doi: 10.62641/aep.v53i6.2035 (PMC12728556; doi:10.62641/aep.v53i6.2035)
Supplement: Supplementary file 1 [file ActEsp-53-6-1354-1365-s1.zip › Supplementary Table 1.docx]

**Supplementary Table 1. Ethics Approval Details of Participating Hospitals**

| **Hospital Name** | **Ethics Committee Approval Number** |
| --- | --- |
| Lu'an Second People's Hospital | LAEY-KYR-CH(20211130) |
| Anqing Sixth People's Hospital | AQLY-JSK-QR(20211203) |
| Huangshan Second People's Hospital | HSEY-JRB-CH(20211109) |
| Xuancheng Fourth People's Hospital | XCSY-HCY-HS(20211212) |
| Feidong County Third People's Hospital | FDSY-LRS-LL(20211206) |
